# Plant and soil nitrogen in oligotrophic boreal forest habitats with varying moss depths: does exclusion of large grazers matter?

**DOI:** 10.1007/s00442-021-04957-0

**Published:** 2021-06-02

**Authors:** Maria Väisänen, Maria Tuomi, Hannah Bailey, Jeffrey M. Welker

**Affiliations:** 1 Ecology and Genetics Research Unit, University of Oulu, Oulu, Finland; 2 Arctic Centre, University of Lapland, Rovaniemi, Finland; 3 Department of Arctic and Marine Biology, UiT The Arctic University of Norway, Tromsø, Norway; 4 Department of Biological Science, University of Alaska Anchorage, Anchorage, AK USA; 5 UArctic, Rovaniemi, Finland

**Keywords:** Reindeer, Dwarf shrub, Inorganic nitrogen, δ^13^C, δ^15^N

## Abstract

The boreal forest consists of drier sunlit and moister-shaded habitats with varying moss abundance. Mosses control vascular plant–soil interactions, yet they all can also be altered by grazers. We determined how 2 decades of reindeer (*Rangifer tarandus*) exclusion affect feather moss (*Pleurozium schreberi*) depth, and the accompanying soil N dynamics (total and dissolvable inorganic N, δ^15^N), plant foliar N, and stable isotopes (δ^15^N, δ^13^C) in two contrasting habitats of an oligotrophic Scots pine forest. The study species were pine seedling (*Pinus sylvestris* L.), bilberry (*Vaccinium myrtillus* L.), lingonberry (*V*. *vitis-idaea* L.), and feather moss. Moss carpet was deeper in shaded than sunlit habitats and increased with grazer exclusion. Humus N content increased in the shade as did humus δ^15^N, which also increased due to exclusion in the sunlit habitats. Exclusion increased inorganic N concentration in the mineral soil. These soil responses were correlated with moss depth. Foliar chemistry varied due to habitat depending on species identity. Pine seedlings showed higher foliar N content and lower foliar δ^15^N in the shaded than in the sunlit habitats, while bilberry had both higher foliar N and δ^15^N in the shade. Thus, foliar δ^15^N values of co-existing species diverged in the shade indicating enhanced N partitioning. We conclude that despite strong grazing-induced shifts in mosses and subtler shifts in soil N, the N dynamics of vascular vegetation remain unchanged. These indicate that plant–soil interactions are resistant to shifts in grazing intensity, a pattern that appears to be common across boreal oligotrophic forests.

**Supplementary Information:**

The online version contains supplementary material available at 10.1007/s00442-021-04957-0.

## Introduction

Across boreal and tundra biomes, mosses contribute to plant biodiversity, biomass, and productivity (Gjerde et al. 2005; Nilsson and Wardle 2005; Cornelissen et al. 2007) and control ecosystem characteristics (Turetsky et al. 2012). For instance, water retaining and insulating mosses control soil microclimate and microclimate-dependent nitrogen (N) mineralization (Gornall et al. 2007; Bernier et al. 2011; Elumeeva et al. 2011; Soudzilovskaia et al. 2013). Mosses also trap N directly from deposition, support biological N fixation and N uptake from soil (DeLuca et al. 2002; Rousk et al. 2013), and supply soil with litter of varying decomposability that may all influence soil N availability, organic matter pools, and, ultimately, vascular vegetation N availability (van der Wal et al. 2001; Bengtsson et al. 2018; Chiapusio et al. 2018; Philben et al. 2018). Additionally, moss effects on ecosystem functions may also vary due to environmental conditions such as changes in air temperature and precipitation (De Long et al. 2016a; Lett et al. 2020). Today, mosses are one of the more susceptible life forms to the on-going global environmental changes (Elmendorf et al. 2012; Fraser et al. 2014; Becker Scarpitta et al. 2017). Since global changes—year-round warming, changes in snow depth and changes in land use—are pronounced and rapidly occurring in the North (IPCC 2019), unravelling the interactions among mosses, their environment, and ecosystem functions is urgent.

Mosses together with lichens and ericaceous dwarf shrubs form the boreal forest (taiga) floor vegetation, which is partly controlled by wildfires and consequent succession where lichens dominate early and mosses and dwarf shrubs later in the successional sequence (Nilsson and Wardle 2005). However, at smaller scales, these plants alternate depending on microclimate: in comparison to mosses and dwarf shrubs, lichens are more abundant in drier microclimatic habitats sustained by soil properties and forest canopy structure (Haughian and Burton 2018; Vitt et al. 2019). Furthermore, mosses and lichens respond to ungulate grazers, such as reindeer (*Rangifer tarandus* L. caribou in North America), whose decimating impacts on lichens are well established across taiga and tundra (Bernes et al. 2015; Köster et al. 2015; Horstkotte and Moen 2019; Uboni et al. 2019). On the contrary, the impact of ungulate grazers on tundra and taiga mosses can range from negative to neutral and positive (Väre et al. 1996; van der Wal and Brooker 2004; Olofsson et al. 2010; Chollet et al. 2013; Bernes et al. 2015; Köster et al. 2015) pointing towards contextual ungulate impact on mosses and, consequently, moss-mediated ecosystem functions.

In boreal forests, locally occurring variations in mosses due to microclimate and grazing may couple with complex changes in ecosystem functions. For instance, ceased grazing could deepen moss carpet, which could in turn enhance N mineralization and thus improve vascular plant growth (Ohtonen and Väre 1998; De Long et al. 2016b; Pacé et al. 2018). Yet, these effects could vary depending on whether deepening mosses have a beneficial (e.g., increased moisture) or unfavorable (e.g., soil cooling) impact on soil microclimate (Väre et al. 1996; van der Wal and Brooker 2004; Olofsson et al. 2010). In addition to the moss-mediated pathways, grazing affects N cycling through nutrient-rich excreta (Bardgett and Wardle 2003) and through trampling that affects soil redox potential and thus N mineralization (Schrama et al. 2013). Indeed, ungulate effects on soil N have been deemed controversial in boreal forests (Stark et al. 2000, 2003; Kolstad et al. 2018). It remains unknown how grazing alters mosses, whether these alterations cascade down to vascular plant–soil interactions in boreal dry and sunlit patches *vs*. moist and shaded habitats, and how these shifts, in turn, compare with the inherent differences due to habitat alone. Resolving these processes could inform on the extent and underlying mechanisms of grazer relative to habitat controls over boreal ecosystem functioning.

Stable N (δ^15^N) and carbon (C, δ^13^C) isotope ratios of plants and soils can reveal the mechanistic interactions between above- and below-ground systems (Dawson et al. 2002; Craine et al. 2009; Elmore et al. 2017). Changes in δ^15^N can reflect differences in soil N availability and plant N acquisition strategies (Welker et al. 2003; Barthelemy et al. 2017). N-rich conditions sustain soil processes (e.g., leaching, gaseous losses) that induce ^15^N enrichment of the soil (inorganic) N pool from which plants draw their N, resulting in foliar δ^15^N enrichment; N-poor conditions sustain more conservative N processing, where N is mostly in organic forms that are ^15^N depleted and lead to depleted foliar δ^15^N values (Högberg 1997; Hobbie and Colpaert 2003). Furthermore, in N-limited systems, plant species differentiate in the timing, depth, and chemical form of N uptake to enable species co-existence (Nadelhoffer et al 1996; McKane et al. 2002). This induces divergence in their foliar δ^15^N composition that may also change due to mycorrhizal symbionts that discriminate the ^15^N isotope differently (Michelsen et al. 1998; Hobbie and Colpaert 2003; Hobbie et al. 2008). Changes in foliar δ^13^C reflect plant water relations, stomatal conductance, and photosynthetic activity that could shift due to altered environmental conditions (Welker et al. 2003; Gavazov et al. 2016). For example, since plant production in boreal forests is often N-limited (Högberg et al. 2017), increased soil N may increase plant N, which boosts photosynthesis enriching foliar δ^13^C values, while drier conditions may also lead to foliar δ^13^C enrichment (Dawson et al. 2002; Wei et al. 2015). In boreal forests, foliar and soil N and C isotopes differ along post-fire successional gradients (Hyodo and Wardle 2009; Hyodo et al. 2013), yet whether habitat and grazers also alter the isotopic signature remains uncertain.

To improve our mechanistic understanding about herbivore–plant–soil interactions in boreal systems, we studied plant and soil characteristics in dry sunlit and moist shaded habitats (herein: “sunlit” and “shaded”) of a boreal pine forest that was either grazed by ungulates or had been protected from ungulates for over 2 decades. Specifically, we test the hypotheses that: (1) Moss carpet is deeper in the shaded than in the sunlit habitats, and deepens by grazer exclusion in both habitats. (2a) Soil N availability and mineralization are greater in the shaded than in the sunlit habitats and they increase by grazer exclusion in both habitats, and (2b) these increases associate with increasing moss depth. (3) Chemical composition varies among plant species, with vascular plant foliar N, δ^15^N, and δ^13^C values increasing in the shaded habitats and after grazer exclusion (higher soil N), whereas mosses exhibit weaker responses, since they are more independent of soil N relative to vascular plants.

## Materials and methods

### Study site

The study site is located in a boreal coniferous forest in northeast Finland, close to the Oulanka research station (66° 37.153′ N, 29° 31.535′ E, 166.5 m a.s.l., Suppl. Fig. S1a). Scots pine (*Pinus sylvestris* L., non-cultivated) is the canopy-forming tree species, which mostly survived from the most recent wildfire phase between 1912 and 1925 (Mickleburgh 2006) and thus forms a mixed-aged forest dominated by older trees. The forest field layer consists of two different habitat types that alternate at 2–5 m distances: (1) the drier, more “sunlit habitat”, dominated by reindeer lichens (*Cladonia* sp.) growing over feather mosses (*Pleurozium schreberi* (Willd. ex Brid.) Mitt.), and (2) the moister, more “shaded habitat”, dominated by feather moss and ericaceous dwarf shrubs (Suppl. Fig. S1b, for vegetation community differences among habitats, see Suppl. Tables S1 and S2). The soil moisture at 5 cm depth was on average 174.3 ± 18.7 mV in the sunlit and 206.7 ± 34.9 mV (mean ± SE) in the shaded habitats during July 2019 (Delta T SM150T). The amount of photosynthetically active radiation (PAR) was 424 ± 335 μmol photons m^−2^ s^−1^ in the sunlit and 341 ± 254 μmol photons m^−2^ s^−1^ (mean ± SE) in the shaded habitats during 22–31 Jul 2019. Soils are freely draining sandy tills and gravels classified as haplic podzol, and the organic horizon, comprising litter and humus, is 0.5–5 cm. The long-term mean annual temperature is − 0.2 °C, July being the warmest (15.0 °C) and January the coldest (− 14.6 °C) month, and the mean annual precipitation is 550.9 mm (1967–2018, Oulanka research station). The semi-domesticated reindeer (*Rangifer tarandus* L.*,* wild caribou in North America) and elk (*Alces alces* L*.*, moose in North America) are common local ungulates with reindeer population density being 1.3 individuals per km^2^ (https://paliskunnat.fi/py/paliskunnat/paliskuntien-tiedot/alakitka/) and elk population density 0.3 individuals per km^2^ (http://riistahavainnot.fi/hirvielaimet/hirvitiheys).

### Grazing treatment and experimental setup

This study was conducted using a long-term fenced area where ungulate grazing has been excluded (hereafter referred to as “fenced”), as well as the adjacent grazed area (hereafter referred to as “grazed”) comprising a similar bedrock, topography, and slope. The fence (2 m high, mesh size 100 × 200 mm) was built in 1994 and covers a 100 × 120 m area. The fence has been meticulously maintained since construction and provides an effective barrier to the local ungulate grazers while allowing smaller grazers, such as hare and voles, to pass through. At the time of our study, the density of pine trees (diam. > 1 cm at 1.3 m height) was 4331 ± 337 trees ha^−1^ in the fenced and 4883 ± 561 trees ha^−1^ (mean ± SE) in the grazed area. The grazing treatment had not affected the composition of the vegetation community (Suppl. Tables S1, S2). Sampling was conducted following a setup that consisted of 12 blocks, six blocks in the fenced area and six blocks in the grazed area, sampled towards different directions around the fence. The distance of the blocks to the fence was ≥ 5 m to omit any fence effects and the distance between blocks was ≥ 20 m. Each block (5 × 5 m) covered both sunlit and shaded habitat that each formed an experimental plot, thus resulting in 24 plots (Suppl. Fig. S1c). The ground vegetation of these plots consisted either of reindeer lichens growing over mosses (sunlit) or solely of mosses (shaded).

### Plant and soil sampling

For foliar chemistry analysis, we sampled pine (*P. sylvestris*) seedlings (15–20 cm height), lingonberry (*Vaccinium vitis-idaea*), and bilberry (*V. myrtillus*) on 9–10 Jul 2018, and on 26–27 Sep 2018, we additionally sampled feather mosses (*P*. *schreberi*). Lingonberry and mosses were found from all plots (*n* = 24), bilberry from 20 plots, and pine seedlings from 15 plots. Whenever the study plant was present in the plot, several shoots of a vascular plant species and several samples of mosses were collected. In September 2018, we also collected reindeer feces from six random locations in the grazed area to be used as a reference of nutrient input levels. After sampling, vascular plant leaves were sorted from stems; for pine seedling and lingonberry, the green fully developed leaves from top of the stem were collected, and green biomass of mosses was separated from brown necromass. Due to logistics, we pooled moss samples by forming pairs of the adjacent spatial blocks but still retaining the grazing and habitat treatments. We considered these pooled moss samples (*n* = 12) to represent yet another six blocks. All samples were air-dried (18 °C).

For soil chemistry analysis, we collected a representative soil sample (11–13 Aug 2018) at each plot (6–12 cores per plot, corer diam. 3 cm) down to a maximum depth of 8 cm after pushing aside mosses. The number of cores varied depending on the depth of humus horizon (Suppl. Table S4), and when very shallow, more cores were needed to secure enough material for analyses. All cores reached mineral horizon, which included a mix of eluviated bleaching and illuviated enrichment horizons. In line with soil sampling, moss layer thickness was measured at 4–9 locations in each plot to an accuracy of 0.1 cm. Lichen layer thickness was not measured as lichens were abundant only in the sunlit habitats and we considered their effect to be integrated with the effect of habitat. Each soil core was split into humus and mineral horizon, and the thickness of horizons was recorded, after which all soil cores were pooled by horizon to a composite humus and a composite mineral soil sample for each plot. Samples were stored cool (+ 5 °C), and organic (2 mm mesh size) and mineral (1.4 mm mesh size) samples were homogenized. Soils were analyzed for moisture (+ 105 °C, 24 h), and loss on ignition (+ 475 °C, 4 h), and soil slurries (10 mL fresh soil, 50 mL MilliQ) were analyzed for pH (Schott-Geräte pH-Meter CG 832) and conductivity (ATI Orion model 170 Conductivity Meter). The known dry weight and sampled soil volumes were used to calculate dry bulk density. Fresh soils were extracted with MilliQ (dry weight-to-total water ratio 1: 52, 2 h, 420 min^−1^), filtered (MN619eh, Macherey Nagel), and analyzed for NH_4_-N and NO_3_-N + NO_2_-N (FIA Lachat) that were summed for total dissolvable inorganic nitrogen (DIN).

### Sample preparation and elemental and stable isotope analyses

The elemental composition and stable isotopes were analyzed from all vascular plant samples (*n* = 59), pooled moss samples (*n* = 12), droppings (*n* = 6), and humus soil (*n* = 24). Plant and dropping samples were dried (+ 50 °C, 72 h) and homogenized into a fine powder (Tissue Lyser). The humus soil samples were dried (+ 50 °C, 24 h) and homogenized into a fine powder with a ball mill. The C and N contents (% dry weight, used to obtain C:N ratios) and stable isotopes, ^13^C and ^15^N, were analyzed with Carlo Erba Flash EA1112 elemental analyzer connected to a Thermo Finnigan DELTA plus Advantage continuous flow stable isotope-ratio mass spectrometer (CF-IRMS). Results are expressed as ratios using the standard δ notation as parts per thousand (‰) difference from the international standards: Vienna Pee Dee belemnite (for C) and atmospheric N_2_ (for N). Precision was always better than 0.06‰ for C and 0.31‰ for N based on the standard deviation of replicates of the standards run repeatedly after every five samples in each sequence.

### Statistical analyses

To test our hypotheses, we employed linear mixed models in R (package *nlme,* Pinheiro et al. 2018) using spatial block as a random intercept to account for the spatial structure of the experimental design. The replication of spatial blocks was 12 for moss depth and soil parameters and 18 for foliar parameters. First, to test the effects of treatments on moss depth (hypothesis 1), we used additive and interactive effects of habitat and grazing as the model fixed terms. To test our second hypothesis, we modelled the additive and interactive effects of habitat and grazing (treatment model) on five soil response variables (humus N%, C:N, δ^15^N, humus and mineral horizon DIN), each in separate models. In addition, we tested how moss depth explained each of these soil response variables. To this end, we fitted models with moss depth as the model fixed term (moss model) and compared treatment models with moss models based on information criteria (Burnham and Anderson 2002; Brewer et al. 2016), and thereafter modelled effects of moss depth on our soil response variables. To test our third hypothesis, we used a three-way interaction of grazing, habitat, and species—*P*. *sylvestris*, *V*. *myrtillus*, *V*. *vitis-idaea,* and moss–as a fixed term to model differences in foliar chemistry (N%, C:N ratio, δ^13^C, δ^15^N). To evaluate significant interactions, we compared species-treatment contrasts using a Tukey’s post hoc test (*emmeans*-package, Lenth 2020). Model fit was confirmed visually by inspecting residual plots, and Log_10_-transformed data were used for soil DIN and foliar C:N ratio to ensure model fit. Full model summary tables for soil and plant chemistry are reported in the electronic supplementary materials (Suppl. Tables S3a, b, S5). Package *ggplot2* (Wickham 2016) was used for data visualizations. All statistical analyses were conducted using the statistical software R (R Development Core Team, version 3.5.0).

## Results

### Moss depth

**Fig. 1:**
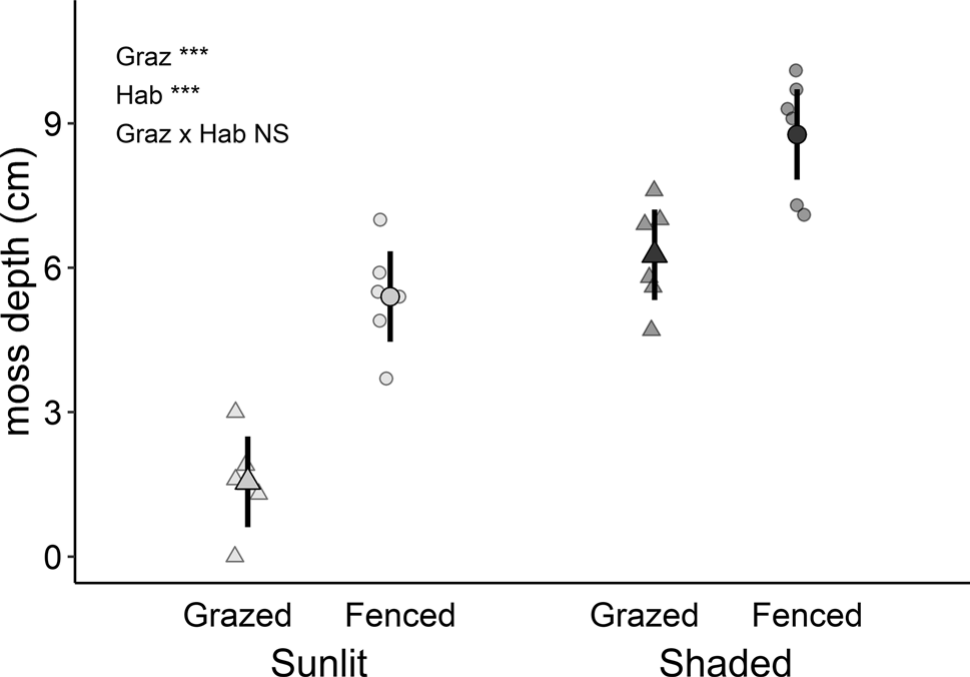
Depth of moss carpet in the boreal forest sunlit (light-grey points) and shaded (dark-grey points) habitats either with (grazed; triangles) or without (fenced; circles) ungulate grazing. Replication for each habitat-grazing combination was six (6). Larger symbols and error bars represent group means with 95% CI estimated by the linear mixed model, whereas smaller symbols indicate observations from individual sampling plots. Significance levels are ***P < 0.0001, *NS* non-significant

Moss depth was twofold in the shaded in comparison to the sunlit habitats (significant effect of habitat, *F*_1,10_ = 96.93, *P* < 0.0001), and grazer exclusion deepened moss carpet 80% compared to the grazed area (significant effect of grazing, *F*_1,10_ = 42.14, *P* = 0.0001, Fig. 1). Effects of habitat and grazing on moss depth did not interact (habitat × grazing–interaction, *F*_1,10_ = 2.703, *P* = 0.1311, Fig. 1).

### Soil chemistry

**Fig. 2:**
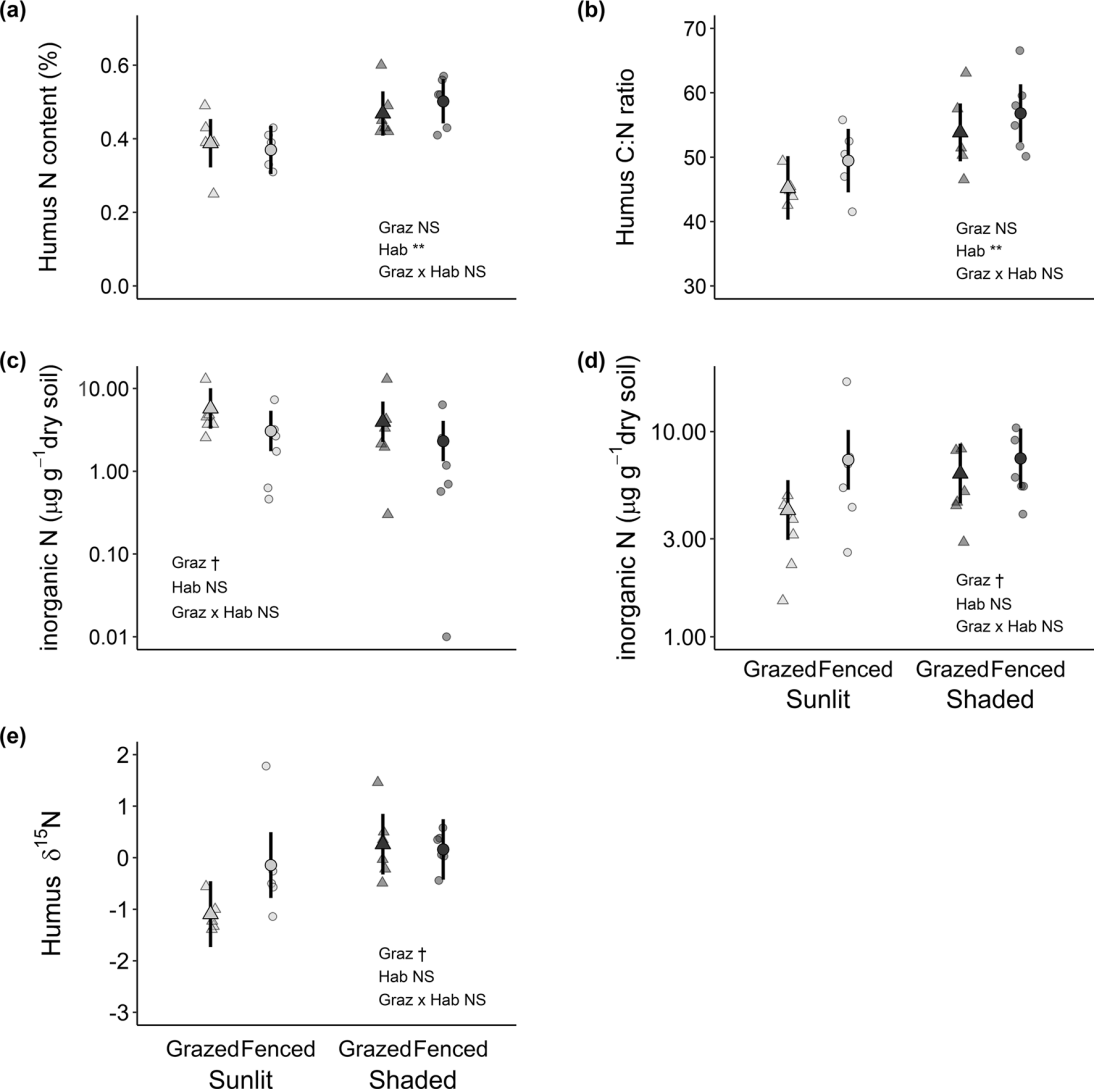
Soil nitrogen in the boreal forest sunlit (light-grey points) and shaded (dark-grey points) habitats either with (grazed; triangles) or without (fenced; circles) ungulate grazing. Larger symbols and error bars represent group means with 95% CI estimated by the linear mixed model, whereas smaller symbols indicate observations from individual sampling plots. **a** Total N content (% dry matter) in the humus horizon, **b** C:N ratio in the humus horizon, **c** inorganic N (the sum of ammonium, nitrate, and nitrite) in the humus and **d** and in the mineral soil horizon, and **e** δ^15^N in the humus horizon. Note a truncated y-axis in panel (**b**), as well as Log10-scaled y-axis in panels (**c**) and (**d**). Significance levels are **P < 0.01, †*P* < 0.100, *NS* non-significant

Humus and mineral horizon N chemistry varied both due to habitat and grazing (Table 1). Humus N was 28% and humus C:N ratio was 17% higher in the shaded than in the sunlit habitats (Table 1, Fig. 2a, b). The concentration of DIN in the humus and the mineral soil horizons did not differ due to habitat but tended to vary due to grazing (Table 1). In the humus horizon, grazing increased DIN twofold, yet with high between-block variability (Fig. 2c, Suppl. Table S3a), whereas in the mineral horizon grazing decreased DIN 32% in comparison to the fenced treatment (Fig. 2d). Humus δ^15^N was more enriched in the shaded than in the sunlit habitats (Table 1). Grazing tended to interact with habitat (*P* = 0.0683, Table 1, Suppl. Table S3a), and humus δ^15^N values were the most depleted in the grazed sunlit habitats while being more similar (enriched) in the fenced sunlit habitats and in the shaded habitats (Fig. 2e). Notably, soil parameters were closely correlated with variation in the moss depth (see Suppl. Table S3b), as all variables except for DIN in humus layer increased significantly with increasing moss depth. Models including moss as the only fixed term performed consistently better compared to models with grazing and habitat treatments, based on information criteria comparison (Suppl. Table S3c). The other soil properties are available in Suppl. Table S4.

**Table 1:** The ANOVA results for nitrogen content (*N*%), carbon-to-nitrogen (C:N) ratio, and δ^15^N in humus horizon and for dissolved inorganic N (DIN, the sum of ammonium, nitrate, and nitrite) in humus and mineral horizon

	Fixed effects	Df	*F* value	*P* value
*N*%	Grazing	1, 10	0.101	0.7572
	Habitat	1, 8	16.02	**0.0039**
	Graz × Hab	1, 8	0.936	0.3616
C:N ratio	Grazing	1, 10	2.490	0.1456
	Habitat	1, 8	12.93	**0.0070**
	Graz × Hab	1, 8	0.114	0.7440
δ^15^N	Grazing	1, 10	1.247	0.2901
	Habitat	1, 8	11.01	**0.0105**
	Graz × Hab	1, 8	4.436	*0.0683*
Log10 (DIN_humus_)	Grazing	1, 10	3.490	*0.0913*
	Habitat	1, 10	2.218	0.1672
	Graz × Hab	1, 10	0.039	0.8459
Log10 (DIN_mineral_)	Grazing	1, 10	3.806	*0.0796*
	Habitat	1, 10	2.823	0.1238
	Graz × Hab	1, 10	2.414	0.1513

Grazing (grazed vs. fenced), habitat (sunlit vs. shaded), and their interactions were used as the fixed terms, and spatial block (*n* = 12) as the random term. The replication was always six for each habitat-grazing combination except for N, C:N ratio, and δ^15^N, which replication was only five for sunlit-grazed and sunlit-fenced treatment combinations. Bold denotes statistically significant (*P* < 0.050) and italics marginally (*P* < 0.100) significant effects. Log_10_-transformed data were used for DIN

### Foliar chemistry

**Fig. 3:**
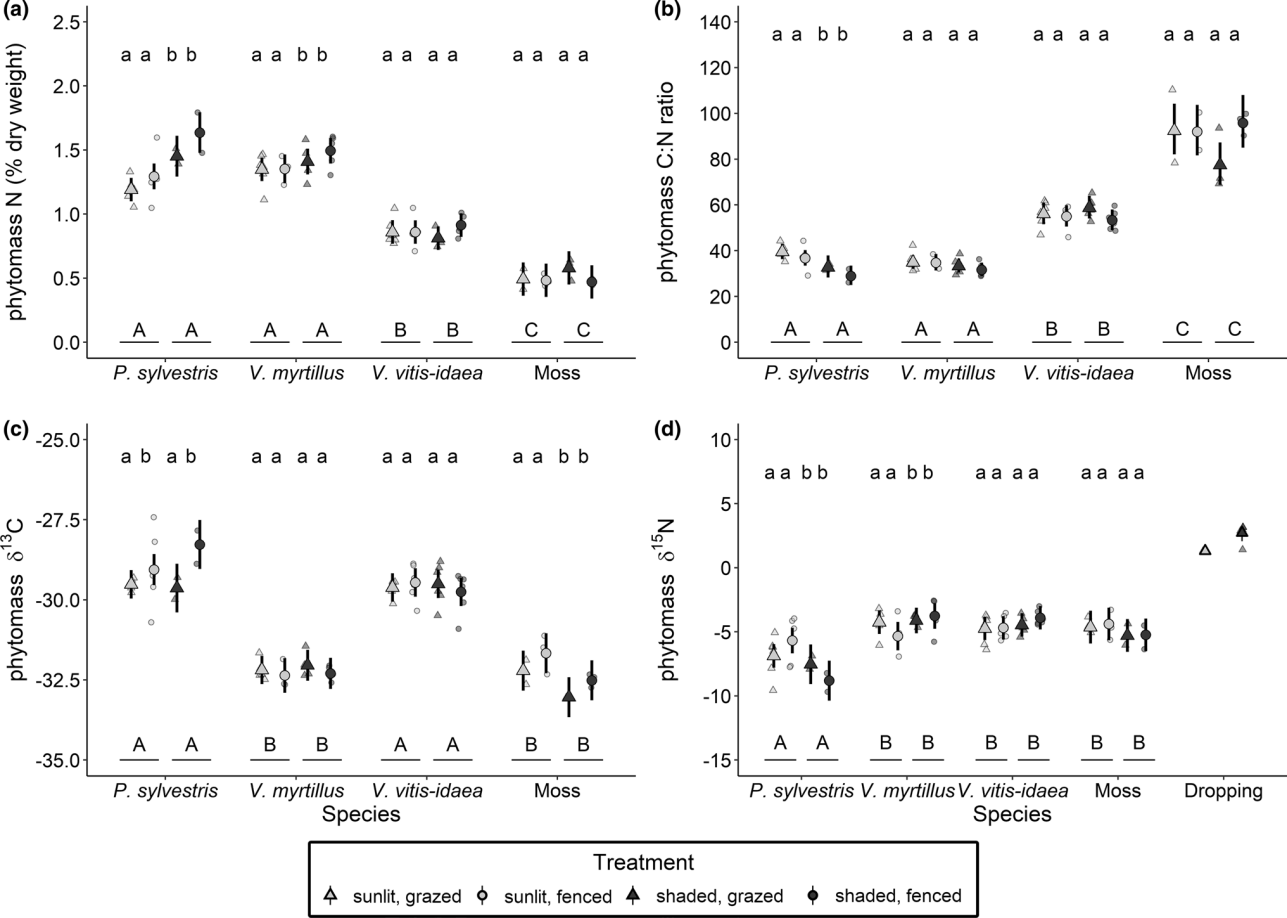
Plant foliar chemistry in the boreal forest sunlit (light-grey points) and shaded (dark-grey points) habitats either with (grazed; triangles) or without (fenced; circles) ungulate grazing. Larger symbols and error bars represent group means with 95% CI estimated by the linear mixed model, whereas smaller symbols indicate observations from individual sampling plots. Foliar **a** N content, **b** C:N ratio, **c** δ^13^C, and **d** δ^15^N of vascular plants and mosses. Note that in (**d**), for reindeer feces, a value of δ^15^N is shown as a reference of N input in sunlit and shaded habitats. The uppercase letters indicate significant (at *P* < 0.05) differences between plant species within the sunlit and the shaded habitats and the lowercase letters indicate significant (at *P* < 0.10) differences due to habitat and grazing within a species, based on post hoc tests

Foliar chemistry varied due to species identity in interaction with habitat (Table 2, Suppl. Table S5). Foliar N content was the lowest in mosses, intermediate in *V*. *vitis*-*idaea*, the highest in *Pinus* seedling and *V. myrtillus*, and varied between the habitats depending on the species (Table 2). Foliar N contents of *Pinus* seedlings and *V*. *myrtillus* were 25 and 7.5% higher in the shaded than in the sunlit habitats, respectively (Fig. 3a). Following the patterns in the foliar N content, foliar C:N ratios were the highest in mosses, intermediate in *V*. *vitis*-*idaea,* and the lowest in *Pinus* seedling and *V*. *myrtillus* and varied between the habitats depending on the species, as the C:N ratio of *Pinus* seedling was 20% lower in the shaded than in the sunlit habitats (Table 2, Fig. 3b). Foliar δ^13^C values varied due to species and were more depleted in *V. myrtillus* and mosses in comparison to *Pinus* seedling and *V*. *vitis-idaea* (Fig. 3c, Table 2). In addition, foliar δ^13^C values showed significant responses to both grazing and habitat depending on the species (Table 2). The foliar δ^13^C values of *Pinus* seedling were more enriched in the fenced than in the grazed treatment especially in the shaded habitat; however, replication was only two for both grazing treatments, questioning the generality of this pattern (Fig. 3c). In addition, the foliar δ^13^C values of mosses were more depleted in the shaded than in the sunlit habitats (Fig. 3c). Foliar δ^15^N values also varied due to species and were more depleted in *Pinus* seedlings in comparison to the other two vascular plant species and mosses that did not differ from each other (Fig. 3d, Table 2). Foliar δ^15^N varied in response to habitat depending on the species, and *Pinus* seedlings had more depleted foliar δ^15^N values in the shaded than in the sunlit habitats, while *V. myrtillus* had more enriched foliar δ^15^N values in the shaded than in the sunlit habitats (Fig. 3d).

**Table 2:** The ANOVA results for the foliar nitrogen content (*N*%), carbon-to-nitrogen (C:N) ratio, and δ^13^C and δ^15^N for plants

	Fixed effects	Df	*F* value	*P* value
*N*%	Grazing	1, 16	0.783	0.3893
	Habitat	1, 39	1.526	0.2242
	Species	3, 39	215.6	**< 0.0001**
	Graz × Hab	1, 39	0.426	0.5179
	Graz × species	3, 39	1.809	0.1615
	Hab × species	3, 39	4.900	**0.0055**
	Graz × Hab × species	3, 39	0.603	0.6170
Log10 (C:N ratio)	Grazing	1, 16	0.03	0.8635
	Habitat	1, 39	0.14	0.7139
	Species	3, 39	280.5	**< 0.0001**
	Graz × Hab	1, 39	0	0.9633
	Graz × species	3, 39	2.30	*0.0919*
	Hab × species	3, 39	2.97	**0.0433**
	Graz × Hab × species	3, 39	1.53	0.2214
δ^13^C	Grazing	1, 16	2.83	0.1122
	Habitat	1, 39	9.93	**0.0031**
	Species	3, 39	161.5	**< 0.0001**
	Graz × Hab	1, 39	0.36	0.5512
	Graz × species	3, 39	2.86	**0.0492**
	Hab × species	3, 39	2.92	**0.0459**
	Graz × Hab × species	3, 39	1.05	0.3818
δ^15^N	Grazing	1, 16	0.096	0.7607
	Habitat	1, 39	1.857	0.1808
	Species	3, 39	18.96	**< 0.0001**
	Graz × Hab	1, 39	0.000	0.9854
	Graz × species	3, 39	0.474	0.7021
	Hab × species	3, 39	4.757	**0.0064**
	Graz × Hab × species	3, 39	2.149	0.1095

Grazing (grazed vs. fenced), habitat (sunlit vs. shaded), species (lingonberry, bilberry, pine seedling, moss), and their interactions were used as the fixed terms, and spatial block (*n* = 18) as the random term. Foliar samples were replicated as follows: lingonberry (*n* = 24), bilberry (*n* = 20), pine seedlings (*n* = 15), and moss (*n* = 12, see Methods for more details). Bold denotes statistically significant (*P* < 0.050) and italics marginally (*P* < 0.100) significant effects. Log_10_-transformed data were used for C:N ratio

## Discussion

We examined how the inherent variation between sunlit and shaded habitats and over 2 decades of ungulate grazer exclusion affected boreal forest’s understory vegetation and soil N dynamics. The magnitude and direction of the impacts of habitat and grazing varied depending on the ecosystem component and measured variable: for instance, the depth of moss carpet responded to both habitat and grazing, soil inorganic N responded to grazer exclusion, while foliar δ^15^N showed species-dependent responses to habitat.

Feather mosses formed deeper carpets in the shaded than in the sunlit habitats and, moss carpets were deeper after grazer exclusion, in support of our first hypothesis. In tundra, exclusion of ungulate grazing also deepens moss carpets most likely due to ceased trampling (Tuomi et al. 2020). In the sunlit habitats, the deepening resulted in moss depths that were nearly the same as in the (grazed) shaded habitats and, thus, sunlit habitats without grazing became more shaded-like with respect to their moss carpet (Fig. 1). It is plausible that grazing-induced declines in moss depth may cascade into moss-mediated soil water availability, and could even amplify the differences that exist among drier and moister microclimatic habitats in (oligotrophic) boreal forests (Haughian and Burton 2018).

Humus N content was higher in the shaded than in the sunlit habitats, whereas excluding grazing increased inorganic N concentration in the mineral soil horizon in both habitats and enriched humus δ^15^N values in the sunlit habitats. These patterns are also associated with deepening moss carpet, thus supporting our second hypothesis that soil N availability and mineralization would increase in the shade as well as in response to grazer exclusion, and that these changes are mediated via mosses. In the shade, the increased humus N content was diluted into greater amount of C (i.e., humus C:N ratio increased); we suggest that this N likely originates from the C-rich moss necromass, which is ample in all but grazed sunlit habitats, if moss depth is used as a proxy of necromass. Consequently, the patterns in the humus C:N ratio closely parallel those in the moss depth as do the patterns in humus δ^15^N (Figs. 1, 2b, e). This latter could stem from the beneficial effects of (deepening) mosses on litter decay and soil microbial N mineralization (Gornall et al. 2007; De Long et al. 2016b). Increasing mineralization sustains processes, such as leaching, that discriminate against ^15^N, thus inducing higher δ^15^N values (Amundson et al. 2003). The higher inorganic N concentration in the mineral horizon due to the grazer exclusion (and moss depth increase) supports the postulate of increased N leaching from the humus. In addition to these moss-linked patterns, grazers increased inorganic N concentration in the humus horizon, plausibly via a direct fertilization effect (Stark and Väisänen 2014). Large grazers may have weak and or site-dependent effects on soil N in boreal forests (Stark et al. 2003; Kolstad et al. 2018). Yet, our findings indicate that at least in dry oligotrophic forests, grazing effects may be easily missed if habitat variation and co-occurring grazer-induced changes in the moss layer are not considered.

Foliar N contents of the ectomycorrhizal evergreen *Pinus* seedling and the ericaceous deciduous dwarf shrub, *V*. *myrtillus*, were higher in the shaded than in the sunlit habitat (Fig. 3a). This may correspond with the higher humus N content observed in the shade, and could also be driven by the deeper moss carpets that benefit N uptake of pine seedlings under harsh conditions (Lett et al. 2017). All vascular plants had depleted foliar δ^15^N values in comparison to humus as found previously (Michelsen et al. 1998; Barthelemy et al. 2017) and caused by their mycorrhizal symbionts that accumulate ^15^N isotope (Hobbie and Colpaert 2003). In addition, foliar δ^15^N values of *Pinus* seedlings and *V. myrtillus* varied with habitat, whereas habitat did not affect δ^15^N values in the ericaceous evergreen dwarf shrub, *V. vitis-idaea*, and non-mycorrhizal mosses (Fig. 3d). These findings partly support our third hypothesis of stronger responses in vascular than non-vascular plants to environmental drivers. The coupled increases in foliar N content and δ^15^N values in *V*. *myrtillus* followed our prediction that higher plants can shift their relative proportions of N sources that are enriched and more abundant in soil (Högberg 1997; Hobbie and Colpaert 2003; Barthelemy et al. 2017). To the contrary, *Pinus* δ^15^N values decreased as foliar N content increased in the shaded (N-rich) habitats. Consequently, the foliar δ^15^N values of these two plant species were more similar in the sunlit than in the shaded habitats. Indeed, boreal trees and dwarf shrubs can have similar foliar δ^15^N values, which suggests similar proportions of divergent N sources (Hyodo et al. 2013). Our findings expand this understanding and suggest that the similarity of plant species’ δ^15^N values in boreal oligotrophic forests may be driven by resource availability and plant uptake strategies and capabilities.

More specifically, plant species δ^15^N values are similar when resources are limited, such as in sunlit habitats where drought limits microbes (Ohtonen and Väre 1998)—including mycorrhizae—and thus reduced mycorrhizae-dependent discrimination of ^15^N (Michelsen et al 1998). Furthermore, shallow humus limits the amount of organic-bound N as well as space for roots and microbes, thus forcing all vascular plants to scavenge for N from the mineral zone that is ^15^N enriched in boreal forests (Lindahl et al. 2007). However, plant species δ^15^N values may start to diverge from each other when resources are not in demand, as in shaded habitats. There, deeper horizons of moss necromass and humus may provide more moisture, space, and a greater proportion of organic-bound N sources in addition to inorganic N (Fig. 2a, c, d) that consequently allow greater variation in the plant’s N acquisition strategies.

In contrast with the foliar N attributes, foliar δ^13^C values of the studied vascular plants did not vary with habitat but only with species, as found previously in boreal forests (Brooks et al. 1997; Hyodo et al. 2013). Compared to vascular plants, the water relations of mosses are more sensitive to environmental factors (Dawson et al. 2002), and indeed, we found mosses to be more enriched in δ^13^C values in the sunlit habitats compared to the shaded, indicative of a drier microclimate and or higher irradiance (Williams and Flanagan 1996; Brooks et al. 1997; Prentice et al. 2011).

To conclude, by considering the inherent sunlit *versus* shaded habitat variation of oligotrophic boreal forest understories, we found that ungulate grazing rivals the role of habitat type in controlling moss layer depth but has minimal direct effects on soil N pools and dynamics. These effects do not cascade down to the N attributes and ecophysiology of understory vascular vegetation, which instead is primarily dictated by species identity and, to a lesser extent, by habitat type. These findings highlight how in addition to large-scale boreal forest fire-induced successional phases (Nilsson and Wardle 2005), smaller scale habitat-driven processes also contribute to plant–soil interactions.

## Supplementary Information

Below is the link to the electronic supplementary material.

Supplementary file1 (PDF 905 kb)

## Data Availability

The datasets including moss depth, all soil parameters, and foliar chemistry are available in figshare with the identifier 10.6084/m9.figshare.14694879.
