# Supplementary material for: Plant and soil nitrogen in oligotrophic boreal forest habitats with varying moss depths: does exclusion of large grazers matter?
Source: Oecologia. 2021 Jun 2;196(3):839–49. doi: 10.1007/s00442-021-04957-0 (PMC8292301; doi:10.1007/s00442-021-04957-0)
Supplement: Supplementary file 1 — Supplementary file1 (PDF 905 kb) [file 442_2021_4957_MOESM1_ESM.pdf]

Electronic Supplementary Materials

Title: Plant and soil nitrogen in oligotrophic boreal forest habitats with varying moss depths: does exclusion of large grazers matter?

Journal: Oecologia

Authors: Maria Väisänen*[1, 2], Maria Tuomi[3], Hannah Bailey[1], Jeffrey M Welker[1, 4, 5]

Affiliations:

1) Ecology and Genetics Research Unit, University of Oulu, Oulu, Finland
2) Arctic Centre, University of Lapland, Rovaniemi, Finland
3) UiT The Arctic University of Norway, Department of Arctic and Marine Biology, Tromsø, Norway
4) University of Alaska Anchorage, Department of Biological Science, Anchorage, AK USA
5) UArctic, Rovaniemi, Finland

*Corresponding author, e-mail: maria.vaisanen@oulu.fi

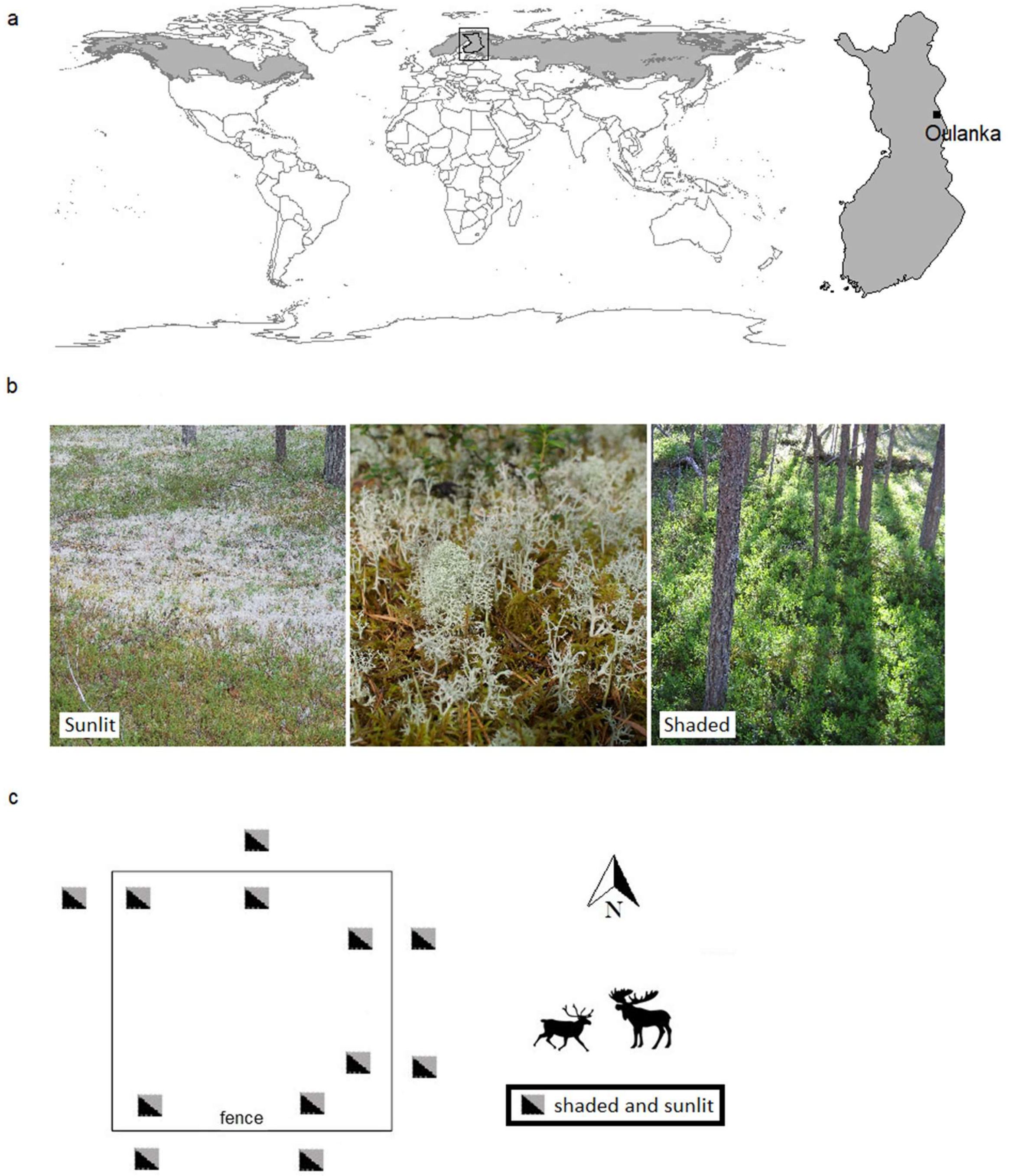

Fig. S1 The sampling area, habitat variation and schematic sampling setup. a) A map showing in grey color the boreal biome. The study site in Finland, Oulanka (66° 37.153′ N, 29° 31.535′ E), is highlighted with a rectangular and enlarged. b) An example showing the inherent variation of vegetation mosaics consisting of drier, more sunlit habitats (“Sunlit”) dominated by reindeer lichens that grow over mosses (the middle panel) and of moister, more shaded habitats

("Shaded") dominated by dwarf shrubs and mosses. These habitats take turns at 2–5 m distances. c) A scheme (not in scale) of the sampling design, which consisted of spatial blocks (filled squares, 5 × 5 m) placed along the fence, six blocks both inside and outside the fence. Each block covered both sunlit (grey) and shaded (black) habitat ("plot"), resulting in altogether 24 experimental plots

Table S1 The abundances of vascular plant species, mosses (feather moss, *Pleurozium schreberi*) and reindeer lichens (*Cladonia* sp.) at the experimental site, Oulanka, northeast Finland (66° 37.153′ N, 29° 31.535′ E, 166.5 m a.s.l.) in July 2018. Vegetation was recorded in open-canopy 'Sunlit' and closed-canopy 'Shaded' habitats in an area grazed by ungulates, 'Grazed', and inside an adjacent fence that had excluded grazing for 24-years, 'Fenced'. Replication was always nine (9) for each habitat and grazing combination. Vegetation was recorded from 50 cm × 40 cm sized areas using a modified point-frequency method (Jonasson 1988[a]) with ten pins and five rows. All hits were recorded and abundances normalized for 100 pins. Values present mean (standard deviation)

**Abundance**	**Sunlit**		**Shaded**	
**(hits 100 pins^{-1})**	Grazed	Fenced	Grazed	Fenced
Calluna vulgaris	33.56 (14.34)	28.00 (27.93)	0.22 (0.67)	0 (0)
Empetrum nigrum	12.22 (6.20)	9.33 (11.79)	20.89 (14.11)	14.44 (10.19)
Vaccinium vitis-idaea	10.89 (4.14)	9.33 (6.78)	22.44 (8.65)	34.22 (13.54)
V. myrtillus	5.78 (11.81)	1.56 (3.13)	31.56 (19.99)	40.67 (10.20)
Pinus sylvestris, seedling	3.33 (2.45)	2.00 (3.32)	6.89 (5.11)	12.89 (15.53)
Mosses	57.33 (34.01)	35.78 (28.57)	98.44 (4.67)	88.89 (6.94)
Lichens	23.56 (17.99)	53.11 (15.88)	3.33 (3.16)	3.33 (4.12)

a Jonasson S (1988) Evaluation of the point intercept method for the estimation of plant biomass. Oikos 52:101–106. https://doi.org/10.2307/3565988

Table S2 The ANOVA results for plant community composition following multivariate and univariate tests. To test the additive and interactive effects of habitat and grazing on vegetation community, we used the *anova.manyglm* function (*mvabund*-package, Wang et al. 2019[b]). Habitat (sunlit *vs*. shaded) and grazing (grazed *vs*. fenced), were used as fixed terms and spatial block (n = 12) was included as a block factor to consider spatial variation. This model used negative binomial distribution, and 999 PIT-trap resampling permutations. Bold denotes statistically significant ($P < 0.050$) and italics marginally ($P < 0.100$) significant effects

Multivariate		Res. Df	Df. diff	Dev	Pr(>Dev)
	Grazing	34	1	6.56	0.587
	Habitat	33	1	130.56	**0.001**
	Graz×Hab	32	1	12.77	0.285
Univariate					
Calluna vulgaris	Grazing			0.051	0.945
	Habitat			42.478	**0.001**
	Graz×Hab			2.132	0.664
Empetrum nigrum	Grazing			1.381	0.807
	Habitat			3.159	0.134
	Graz×Hab			0.035	0.872
Vaccinium myrtillus	Grazing			0.038	0.945
	Habitat			14.42	**0.017**
	Graz×Hab			2.556	0.664
Vaccinium vitis-idaea	Grazing			1.256	0.807
	Habitat			23.741	**0.006**
	Graz×Hab			3.033	0.620
Pinus sylvestris	Grazing			0.827	0.807
	Habitat			10.047	**0.033**
	Graz×Hab			2.564	0.664
Mosses	Grazing			1.164	0.807
	Habitat			14.799	**0.017**
	Graz×Hab			1.256	0.664
Lichens	Grazing			1.842	0.735
	Habitat			21.917	**0.006**
	Graz×Hab			1.19	0.664

b Wang Y, Naumann U, Eddelbuettel D, Wilshire J, Warton D (2019) mvabund: Statistical methods for analysing multivariate abundance data. R package version 4.0.1. https://CRAN.R-project.org/package=mvabund

Table S3a Results of mixed effects models on soil variables, where fixed effects include additive and interactive effects of grazing ("grazed" *vs*. "fenced") and habitat ("sunlit" *vs*. "shaded" and spatial block (n = 12) was used as a random factor. Models were fitted using restricted maximum likelihood estimation. Statistically significant results ($P < 0.05$) are indicated in bold, and results where $0.05 < P < 0.1$ and with large model estimates are indicated in italics. Log_{10}-transformed data was used for dissolved inorganic nitrogen (DIN) in humus and mineral soil horizons

response	fixed term	estimate	std.error	DF	t-value	*P*-value
N%	Intercept (fenced : sunlit)	0.369765	0.031245	10	11.83452	<0.001
	grazed	0.018002	0.044187	10	0.40741	0.692
	shaded	0.131902	0.037654	8	3.503004	**0.008**
	grazed : shaded	-0.05134	0.053251	8	-0.96403	0.363
C: N ratio	Intercept (fenced: sunlit)	49.45066	2.344767	10	21.0898	<0.001
	grazed	-4.23472	3.316001	10	-1.27706	0.230
	shaded	7.35956	3.174829	8	2.318095	**0.049**
	grazed : shaded	1.2539	4.489887	8	0.279273	0.787
$\delta^{15}N$	Intercept (fenced : sunlit)	-0.14369	0.303931	10	-0.47276	0.647
	grazed	-0.95329	0.429824	10	-2.21786	*0.051*
	shaded	0.303687	0.355003	8	0.855449	0.417
	grazed : shaded	1.054956	0.50205	8	2.101295	*0.069*
Log10 (DIN_{humus})	Intercept (fenced : sunlit)	0.486772	0.11691	10	4.163642	0.002
	grazed	0.271368	0.165336	10	1.641311	0.132
	shaded	-0.12301	0.134848	10	-0.91224	0.383
	grazed : shaded	-0.03803	0.190704	10	-0.19943	0.846
Log10 ($DIN_{mineral}$)	Intercept (fenced : sunlit)	0.862235	0.069751	10	12.36166	0
	grazed	-0.24524	0.098642	10	-2.48618	**0.032**
	shaded	0.006971	0.077944	10	0.089437	0.931
	grazed : shaded	0.171272	0.110229	10	1.553779	0.151

Table S3b Results of mixed effects models on soil variables, where fixed effects include only moss depth as a continuous variable and random effects included spatial block (n = 12) as an intercept. Models were fitted using restricted maximum likelihood estimation. Statistically significant results ($P < 0.05$) are indicated in bold. Log_{10}-transformed data was used for dissolved inorganic nitrogen (DIN) in humus and mineral soil horizons

response	fixed term	estimate	std.error	DF	t-value	p-value
N%	Intercept	0.333981	0.036864	11	9.05975	<0.001
	Moss depth (cm)	0.018231	0.005809	9	3.138668	**0.012**
C: N ratio	Intercept	43.38293	2.747181	11	15.7918	<0.001
	Moss depth (cm)	1.46314	0.438984	9	3.333019	**0.009**
$\delta^{15}N$	Intercept	-1.25809	0.348362	11	-3.61143	0.004
	Moss depth (cm)	0.191537	0.053855	9	3.556511	**0.006**
Log10 (DIN_{humus})	Intercept	0.74877	0.12591	11	5.946871	<0.001
	Moss depth (cm)	-0.03591	0.019285	11	-1.86187	0.090
Log10 ($DIN_{mineral}$)	Intercept	0.63167	0.075583	11	8.357308	<0.001
	Moss depth (cm)	0.028066	0.011577	11	2.424209	**0.034**

Table S3c Information criteria comparisons of linear mixed effects models with alternative fixed terms, where a smaller value indicates a more parsimonious model, and where increasing difference in the models' ICs indicates increasing support (Burnham and Anderson 2002[a]; Richards et al. 2011[b]; Brewer et al. 2016[c]). If difference in ICs is < 2, neither model is considerably better. Grazing × Habitat correlates strongly with variation in moss depth, and thus these two sets of variables were not included in the same model. For model comparison, models were fitted using maximum likelihood estimation

response	fixed term	random term	df	AICc	BIC
N%	Grazing×Habitat	1|Repl.block	6	-41.82596	-40.8797
	Moss depth (cm)		4	-44.83144	-42.8202
C:N ratio	Grazing×Habitat	1|Repl.block	6	148.5224	149.4686
	Moss depth (cm)		4	145.0619	147.0732
$\delta^{15}N$	Grazing×Habitat	1|Repl.block	6	58.07922	59.02548
	Moss depth (cm)		4	53.93006	55.94129
Log10 (DIN_{humus})	Grazing×Habitat	1|Repl.block	6	19.22537	21.35252
	Moss depth (cm)		4	14.93256	17.53951
Log10 ($DIN_{mineral}$)	Grazing×Habitat	1|Repl.block	6	-5.964253	-3.83711
	Moss depth (cm)		4	-9.563977	-6.95703

a Burnham KP, Anderson DR (2002) Model Selection and Multimodel Inference: A Practical Information-Theoretic Approach, 2nd edn. Springer, Berlin.

b Richards SA, Whittingham MJ, Stephens PA (2011) Model selection and model averaging in behavioural ecology: the utility of the IT-AIC framework. Behav Ecol Sociobiol 65:77–89. https://doi.org/10.1007/s00265-010-1035-8

c Brewer MJ, Butler A, Cooksley SL (2016) The relative performance of AIC, AICC and BIC in the presence of unobserved heterogeneity. Methods Ecol Evol 7: 679–692. http://doi.org/10.1111/2041-210X.12541

Table S4 Selected properties of the humus and mineral soil horizon at the experimental site, Oulanka, northeast Finland (66° 37.153′ N, 29° 31.535′ E, 166.5 m a.s.l.). The experimental site was a boreal oligotrophic Scots pine forest consisting of open canopy habitats ("Sunlit"), and closed canopy habitats ("Shaded"). The grazing treatment consisted of a grazed ("Grazed") area and a long-term ungrazed ("Fenced") area. Values present mean (standard deviation) and the number of replicates for each habitat and grazing treatment combination was six for all parameters except for $\delta^{13}C$ for which replication was five for sunlit-grazed and sunlit-fenced treatment combinations due to analytical failure

	Sunlit		Shaded	
	Grazed	*Fenced*	*Grazed*	*Fenced*
Humus				
Humus depth (cm)	0.97 (0.30)	1.13 (0.27)	2.68 (0.61)	2.60 (0.57)
Bulk density (g cm^{-3})	0.36 (0.12)	0.26 (0.05)	0.18 (0.03)	0.14 (0.03)
Moisture%	40.05 (7.95)	40.88 (4.53)	47.5 (3.51)	54.21 (4.96)
OM%	32.27 (9.39)	30.35 (4.88)	40.85 (5.65)	48.98 (4.48)
Conductivity (µS cm^{-1})	18.97 (6.10)	23.65 (7.21)	32.73 (6.81)	29.1 (5.40)
pH	3.67 (0.14)	3.75 (0.11)	3.46 (0.09)	3.61 (0.05)
$\delta^{13}C$	-28.34 (0.49)	-28.26 (0.54)	-28.47 (0.19)	-28.35 (0.21)
Mineral				
Bulk density (g cm^{-3})	0.95 (0.20)	0.87 (0.08)	0.93 (0.11)	0.75 (0.13)
Moisture%	11.92 (3.30)	11.45 (2.16)	13.7 (1.61)	15.02 (1.84)
OM%	3.45 (1.12)	2.89 (0.92)	3.01 (0.89)	4.44 (1.10)
Conductivity (µS cm^{-1})	9.57 (2.81)	11.21 (4.45)	14.73 (2.43)	16.01 (2.52)
pH	3.89 (0.25)	3.94 (0.16)	3.67 (0.12)	3.75 (0.08)

Table S5 Results of mixed effects models on plant variables, where fixed effects include additive and interactive effects of grazing ("grazed" *vs*. "fenced"), habitat ("sunlit" *vs*. "shaded") and species *Pinus* seedling ("Pinus"), *Vaccinium myrtillus* ("Vmyr"), *V. vitis-idaea* ("Vvit") and moss ("moss"). Spatial block (n = 18) was used as a random factor. Models were fitted using restricted maximum likelihood estimation. Statistically significant results ($P < 0.05$) are indicated in bold, and results where $0.05 < P < 0.1$ and with large model estimates are indicated in italics. Log_{10}-transformed data was used for C: N ratio

response	fixed term	estimate	std.error	DF	t-value	*P*-value
N%	Intercept (fenced : sunlit : moss)	0.483238	0.064637	39	0.06463	<0.001
	grazed	0.009241	0.091411	16	0.09141	0.9207
	shaded	-0.013032	0.091411	39	-0.14256	0.8874
	Pinus	0.810655	0.081760	39	9.91495	**<0.001**
	Vmyr	0.869740	0.085507	39	10.17150	**<0.001**
	Vvit	0.376050	0.079164	39	4.75022	**<0.001**
	grazed : shaded	0.100148	0.129275	39	0.77469	0.4432
	grazed : Pinus	-0.112383	0.113806	39	-0.98749	0.3295
	grazed : Vmyr	-0.013353	0.116527	39	-0.11459	0.9094
	grazed : Vvit	-0.008603	0.111955	39	-0.07685	0.9391
	shaded : Pinus	0.354843	0.130881	39	2.71118	**0.0099**
	shaded : Vmyr	0.154324	0.118306	39	1.30445	0.1997
	shaded : Vvit	0.067534	0.111955	39	0.60322	0.5498
	grazed : shaded : Pinus	-0.181252	0.183961	39	-0.98527	0.3306
	grazed : shaded : Vmyr	-0.180280	0.164159	39	-1.09820	0.2788
	grazed : shaded : Vvit	-0.202436	0.158329	39	-1.27858	0.2086
Log10 (C:N ratio)	Intercept (fenced : sunlit : moss)	1.963778	0.025905	39	75.80637	<0.001
	grazed	0.002279	0.036635	16	0.06223	0.9512
	shaded	0.017765	0.036635	39	0.48494	0.6304
	Pinus	-0.399022	0.032767	39	-12.17729	**<0.001**
	Vmyr	-0.422721	0.034269	39	-12.33526	**<0.001**
	Vvit	-0.223239	0.031727	39	-7.03622	**<0.001**
	grazed : shaded	-0.094708	0.051810	39	-1.82797	0.0752
	grazed : Pinus	0.029700	0.045610	39	0.65117	0.5188
	grazed : Vmyr	-0.000750	0.046701	39	-0.01606	0.9873
	grazed : Vvit	0.006164	0.044869	39	0.13738	0.8914
	shaded : Pinus	-0.121257	0.052454	39	-2.31168	**0.0262**
	shaded : Vmyr	-0.058288	0.047414	39	-1.22933	0.2263
	shaded : Vvit	-0.031698	0.044869	39	-0.70645	0.4841
	grazed : shaded : Pinus	0.116733	0.073727	39	1.58331	0.1214
	grazed : shaded : Vmyr	0.115166	0.065791	39	1.75048	*0.0879*
	grazed : shaded : Vvit	0.129062	0.063454	39	2.03394	**0.0488**

Table S5 continued

response	fixed term	estimate	std.error	DF	t-value	*P*-value
	Intercept (fenced : sunlit : moss)	-31.64597	0.3116871	39	-101.53120	<0.001
	grazed	-0.58533	0.4407921	16	-1.32791	0.2028
	shaded	-0.84872	0.4248408	39	-1.99773	0.0528
	Pinus	2.58476	0.3940338	39	6.55974	**<0.001**
	Vmyr	-0.71536	0.4118446	39	-1.73698	0.0903
	Vvit	2.18626	0.3817372	39	5.72714	**<0.001**
	grazed : shaded	0.01915	0.6008156	39	0.03187	0.9747
$\delta^{13}C$	grazed : Pinus	0.12951	0.5486218	39	0.23607	0.8146
	grazed : Vmyr	0.75662	0.5615508	39	1.34738	0.1856
	grazed : Vvit	0.42924	0.5398579	39	0.79511	0.4314
	shaded : Pinus	1.64536	0.6147850	39	2.67632	**0.0108**
	shaded : Vmyr	0.91251	0.5505587	39	1.65743	0.1055
	shaded : Vvit	0.55441	0.5203216	39	1.06552	0.2932
	grazed : shaded : Pinus	-0.93534	0.8625330	39	-1.08441	0.2848
	grazed : shaded : Vmyr	0.07316	0.7638080	39	0.09579	0.9242
	grazed : shaded : Vvit	0.38902	0.7358459	39	0.52867	0.6000
	Intercept (fenced : sunlit : moss)	-4.521259	0.644310	39	-7.01720	<0.001
	grazed	0.021631	0.911192	16	0.02374	0.9814
	shaded	-0.845681	0.863829	39	-0.97899	0.3336
	Pinus	-1.150777	0.814133	39	-1.41349	0.1654
	Vmyr	-0.781662	0.850468	39	-0.91909	0.3637
	Vvit	-0.167486	0.789116	39	-0.21224	0.8330
	grazed : shaded	0.194136	1.221639	39	0.15891	0.8746
$\delta^{15}N$	grazed : Pinus	-1.228524	1.133806	39	-1.08353	0.2852
	grazed : Vmyr	1.033756	1.160172	39	0.89103	0.3784
	grazed: Vvit	-0.070831	1.115978	39	-0.06346	0.9497
	shaded : Pinus	-2.325280	1.254350	39	-1.85377	*0.0713*
	shaded : Vmyr	2.393477	1.119931	39	2.13716	**0.0389**
	shaded : Vvit	1.634008	1.057970	39	1.54447	0.1306
	grazed : shaded : Pinus	2.365925	1.758663	39	1.34529	0.1863
	grazed : shaded : Vmyr	-1.562543	1.553626	39	-1.005739	0.3207
	grazed : shaded : Vvit	-0.705919	1.496196	39	-0.47180	0.6397
